# Worst-Case Robustness Evaluation Methods for IMPT: A Critical Comparison

**DOI:** 10.1016/j.ijpt.2025.101199

**Published:** 2025-08-05

**Authors:** Chunbo Liu, Chris J. Beltran, Jiajian Shen, Niles Zhang, Yifei Pi, Martin Bues, Justin Park, Bo Lu, Sridhar Yaddanapudi, Jun Tan, Keith M. Furutani, Xiaoying Liang

**Affiliations:** a Department of Radiation Oncology, The First Affiliated Hospital of Zhengzhou University, Zhengzhou, China; b Department of Radiation Oncology, Mayo Clinic, Jacksonville, Florida, USA; c Department of Radiation Oncology, Mayo Clinic, Phoenix, Arizona, USA

**Keywords:** Intensity-modulated proton therapy, Robustness evaluation, Worst-case approach

## Abstract

**Purpose:**

Robustness evaluation is routinely used in clinics to ensure the intended dose delivery for intensity-modulated proton therapy (IMPT). Various methods have been proposed, but there is no consensus on which method should be adopted in clinical practice. This study examined various methods within the widely used worst-case approach to provide insights into IMPT plan evaluation.

**Materials and Methods:**

We evaluated the robustness of 20 clinical IMPT plans (10 prostate and 10 head and neck). Five robustness evaluation methods were assessed: error-bar dose distribution (ebDD), root-mean-square error dose (RMSED) distribution, voxel-wise worst-case, physical scenario worst-case, and dose-volume histogram (DVH) band. Correlations between these methods were analyzed. Each method was reviewed for their quantitative and qualitative capabilities to identify potential underdosing or overdosing.

**Results:**

Strong correlations were found between ebDD and RMSED, and between voxel-wise worst-case and physical scenario worst-case. The DVH band method provides a straightforward way to assess whether the worst DVH meets plan criteria and to illustrate dose variations but lacks spatial detail to pinpoint areas of potential underdosing or overdosing. The voxel-wise worst-case captures the worst dose distribution across all evaluation metrics, allowing spatial identification of areas of concern within a single distribution. The physical scenario worst-case also pinpoints specific areas of concern but requires individual assessment for each region of interest and evaluation metric, which can be cumbersome. A 3D visualization with ebDD and RMSED highlights regions of dose variation but does not necessarily indicate clinically meaningful impact.

**Conclusion:**

Different robustness evaluation methods offer different types of information. Our study provides valuable insights to help identify an effective and practical approach for clinical practice. Based on our findings, we propose a potential evaluation strategy: use the DVH band derived from physical uncertainty scenarios to assess whether the worst boundary values meet plan evaluation criteria, and, when concerns arise, apply the voxel-wise worst-case dose distribution to localize areas of potential risk.

## Introduction

Robustness evaluations are essential for intensity-modulated proton therapy (IMPT) plans to ensure that the intended dose is delivered under uncertainty scenarios, such as setup and range uncertainties. In recent years, robustness evaluation has been a significant research focus, leading to the development of various methods. These methods generally fall into 2 categories: the worst-case approach^1^, ^2^, ^3^, ^4^, ^5^, ^6^, ^7^ and probabilistic approach.^8^, ^9^, ^10^, ^11^, ^12^, ^13^

The probabilistic approach involves sampling both random and systematic errors across hundreds of uncertainty scenarios, typically assuming that errors follow a normal distribution, to generate a range of possible doses along with a confidence interval. Given the substantial computational cost and time required for performing several hundred dose calculations per plan, various methods have been proposed to achieve fast yet accurate dose predictions. These methods include fast dose calculation techniques based on precomputed nominal dose distributions while accounting for changes in the radiologic path length due to setup and range uncertainties,^9^ the development of a meta-model that approximates the dose distribution using polynomial chaos expansion,^13^ and the application of deep learning for dose distribution prediction.^12^

The worst-case analysis, initially proposed by Goitein,^14^ suggested that, in addition to evaluate the dose in the nominal scenario, the dose in the “worst-case” should also be considered. Today, the worst-case analysis that assumes systematic errors is widely used in clinics to model the effect of delivery uncertainties in proton therapy. Typically, a limited number of uncertainty scenarios, usually between 8 and 21, are sampled to assess the dose distribution under setup and range variations.^15^ These scenarios represent a discrete set of clinically relevant perturbations derived from the underlying uncertainties, such as setup and range variations. Each scenario is simulated by recalculating the dose distribution, incorporating rigid patient shifts relative to the isocenter and scaled Hounsfield unit values of the computed tomography images. Within the worst-case framework, it is further divided into voxel-wise and physical scenario worst-case.^16^ The voxel-wise worst-case represents the worst dose value for each voxel when errors are introduced.^7^, ^17^ Since the worst dose value for different voxels may come from various uncertainty scenarios, this distribution is unlikely to reflect a physically realizable scenario. The physical scenario worst-case maintains voxel correlation, meaning that the doses in all voxels originate from the same physically realizable uncertainty scenario.^18^

For quantitative or qualitative evaluation using the worst-case approach, various methods and metrics have been proposed. Albertini et al^1^ introduced the error-bar dose distribution (ebDD), which represents the range of dose values at each voxel across different scenarios, visually highlighting areas of significant dose variation due to uncertainties.^1^, ^19^ Similarly, Liu et al^3^ proposed the root-mean-square error dose (RMSED) per voxel. Both the ebDD and RMSED methods provide voxel-wise evaluations, offering spatial information on where significant dose variations might occur due to uncertainties. In an effort to find a practical approach for proton plan assessment consistent with PTV evaluations used in photon therapy, Korevaar et al^2^ compared voxel-wise minimum, voxel-wise mean, and worst-case scenario methods. They recommended using voxel-wise minimum D98 for target coverage evaluation and voxel-wise maximum D2 for hotspot evaluation. Another branch of evaluation methods is based on dose-volume histogram (DVH). DVH-based evaluation involves computing DVH curves for each uncertainty scenario and displaying the variability of a family of DVH curves to visualize the envelope of DVH for various scenarios.^4^, ^6^, ^20^

A recent seminal systematic review^15^ showed that the setup and range uncertainty parameters used for robustness evaluation are relatively consistent across centers, with the worst-case approach being the most commonly adopted method. However, there remains a lack of harmonization in evaluation methods within the framework of the worst-case approach.^15^ This finding aligns with the survey study by Kaplan et al,^21^ which showed that robustness evaluation methods vary across centers and that there is currently no consensus on a standardized approach.

In this study, we systematically compared existing methods within the widely adopted worst-case approach and examined their respective strengths and limitations. Specifically, we evaluated the robustness of 20 clinical IMPT plans using 5 different methods: ebDD, RMSED, voxel-wise worst-case, physical scenario worst-case, and DVH band. The goal of this work is to provide informative data to aid in the selection of robustness evaluation strategies in clinical practice.

## Materials and Methods

This study was granted Institutional Review Board exemption by the Mayo Clinic Institutional Review Board (No.: 22-009186).

Ten consecutive prostate and 10 consecutive head and neck clinical IMPT plans were included in the study. The treatment plans were optimized using the Eclipse treatment planning system (TPS), version 15.6 (Varian Medical Systems). All plans were robustly optimized on the clinical target volume (CTV) using setup uncertainties of 3 mm for head and neck and 5 mm for prostate, along with a 3.5% range uncertainty. Organ at risk (OAR) optimization was performed on the nominal scenario only. Target coverage was assessed by the minimum dose received by 95% of the CTV volume (CTV D95%). For prostate plans, the V90% and V65% for bladder and rectum were evaluated. For head and neck plans, OAR evaluation metrics included optic chiasm D0.01cc, spinal cord D0.01cc, brain stem D0.01cc, parotid Dmean, constrictor Dmean, larynx Dmean, and oral cavity Dmean.

Dose distributions were recalculated in the TPS for 12 uncertainty scenarios, each combining setup uncertainties (3 mm for head and neck cases, 5 mm for prostate cases) in different directions with a 3.5% range uncertainty, as detailed in Table 1.

**Table 1:** Setup and range uncertainty settings for robustness evaluation for 12 scenarios (U1-U12).

Uncertainty scenarios	Setup uncertainty (mm)			Range uncertainty (%)
	L/R	A/P	S/I	
U1	3 or 5	0	0	3.5
U2	3 or 5	0	0	−3.5
U3	−3 or −5	0	0	3.5
U4	−3 or −5	0	0	−3.5
U5	0	3 or 5	0	3.5
U6	0	3 or 5	0	−3.5
U7	0	−3 or −5	0	3.5
U8	0	−3 or −5	0	−3.5
U9	0	0	3 or 5	3.5
U10	0	0	3 or 5	−3.5
U11	0	0	−3 or −5	3.5
U12	0	0	−3 or −5	−3.5

5 mm setup uncertainty for prostate and 3 mm setup uncertainty for head and neck cases.

The 3D dose matrices of the nominal scenario and the 12 uncertainty scenarios were post processed to obtain the 3D matrices of ebDD, RMSED, voxel-wise worst-case (voxel-wise Min for target coverage and voxel-wise Max for OARs), as detailed below for each method.

For each voxel,

$$\mathrm{ebDD}\left(x,y,z\right)=\max_{i=0}^{12}D_{i}\left(x,y,z\right)-\min_{i=0}^{12}D_{i}\left(x,y,z\right)$$

$$\mathrm{RMSED}\left(x,y,z\right)=\sqrt{\frac{\sum_{i=1}^{12}{\left(D_{0}\left(x,y,z\right)-D_{i}\left(x,y,z\right)\right)}^{2}}{12}}$$

$$\mathrm{VoxelwiseMin}\left(x,y,z\right)=\min_{i=0}^{12}D_{i}\left(x,y,z\right)$$

$$\mathrm{VoxelwiseMax}\left(x,y,z\right)=\max_{i=0}^{12}D_{i}\left(x,y,z\right)$$

where $D_{i}(x,y,z)$ represents the dose at voxel (x,y,z) in scenario i, with i ranging from 0 to 12. While the index i=0represents the nominal scenarios, and the index of i=1to12represent the 12 uncertainty scenarios.

In addition to the ebDD, RMSED, and voxel-wise worst-case, we also evaluated the physical scenario worst-case across the nominal and the U1 to U12 scenarios. The ebDD, RMSED, voxel-wise worst-case, and physical scenario worst-case methods contain 3D data matrices that can be visualized for qualitative evaluation. The voxel-wise worst-case and physical scenario worst-case methods display the dose for each voxel, while the ebDD and RMSED methods show dose variations for each voxel.

For quantitative evaluation, the error-bar volume histogram (EVH) and RMSED volume histogram (RVH) were generated from the 3D dose matrices of ebDD and RMSED for each region of interest (ROI). The area under the curve (AUC) of EVH and RVH for each investigated ROI was calculated. A smaller AUC value indicates less variation across the scenarios. In total, 35 CTV volumes and 81 OAR ROIs were included. Additionally, the family of DVHs across the nominal and U1-U12 scenarios for each investigated ROI was generated, and the bandwidth at each DVH evaluation metric point was used to quantitatively assess the variation. The DVH-point values for each evaluation metric were also extracted from the voxel-wise worst-case and physical scenario worst-case approaches. A total of 35 CTV D95% values and 101 DVH points for OARs were included.

Pearson correlation analysis was performed to evaluate the correlation of the evaluation methods.

## Results

### Correlation analysis

Table 2 lists the Pearson correlation coefficients and *P*-values for the different robustness evaluation methods.

**Table 2:** Pearson correlation coefficients and *P*-values for different robustness evaluation methods.

	EVH_AUC	RVH_AUC	Bandwidth	Voxel-wise worst-case	Physical scenario worst-case
CTV					
EVH AUC	1				
RVH AUC	0.99, <<0.01	1			
Bandwidth	0.66, <<0.01	0.67, <<0.01	1		
Voxel-wise worst-case	−0.24, 0.16	−0.25, 0.14	−0.64, <<0.01	1	
Physical scenario worst-case	−0.20, 0.25	−0.21, 0.22	−0.65, <<0.01	0.92, <<0.01	1
OARs					
EVH AUC	1				
RVH AUC	0.99, <<0.01	1			
Bandwidth	0.51, <<0.01	0.49, <<0.01	1		
Voxel-wise worst-case	0.25, 0.01	0.23, 0.02	0.31, 0.001	1	
Physical scenario worst-case	0.23, 0.03	0.21, 0.04	0.32, 0.001	1, <<0.01	1

**Abbreviations: EVH, error-bar volume histogram; AUC, area under the curve; RVH, RMSED volume histogram**.

Amony the 5 robustness evaluation methods, the AUC of EVH generated from ebDD and the AUC of RVH generated from RMSED were strongly correlated, with *r* = 0.99 and *P* << .01 for both CTV and OAR. Similarly, voxel-wise and physical scenario worst-case evaluations were strongly correlated (*r* = 0.92 and 0.998 for CTV and OARs, respectively, both with *P* << .01). The DVH bandwidth showed moderate correlations with the other 4 methods.

### Dosimetric quantitative evaluation

**Figure 1:**
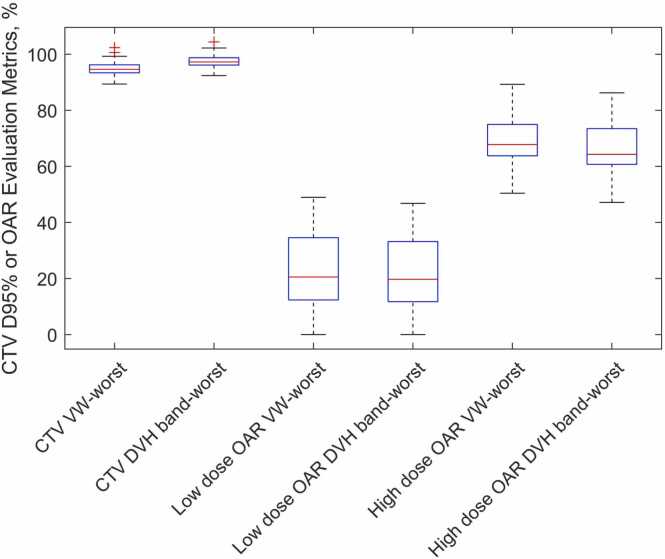
Box plot comparison of DVH-point values between the voxel-wise worst-case (VW-Worst) and the worst value in the DVH band derived from physical uncertainty scenarios (DVH band-worst) for the CTV and OARs. Dose is expressed as a percentage of the prescription. The OARs were divided into a low-dose group (<50% Rx) and a high-dose group (≥50% Rx) to aid in the interpretation of the data. Each box shows the interquartile range, with the line inside representing the median value. Abbreviation: DVH, dose-volume histogram.

Figure 1 shows the box plot of the CTV D95% and relevant OAR evaluation metrics. To provide a more meaningful presentation, the dose is expressed as a percentage of the prescription dose (Rx), given the wide range of prescriptions in our cohort. Additionally, the OARs were divided into a low-dose group (<50% Rx) and a high-dose group (≥50% Rx) to aid in the interpretation of the data. In Figure 1, we observe that for CTV D95%, the values obtained from the voxel-wise worst-case were more conservative (ie, lower) than those from the DVH band derived from the physical scenario worst-case, with a median difference of 2.5% (range: 0.2%-4.2%). A similar trend was seen for high-dose OARs, where voxel-wise worst-case values were more conservative (ie, higher). For low-dose OARs, both the voxel-wise worst-case and DVH band-worst values yielded similar results, as the low-dose regions are less affected by delivery uncertainties.

### 3D visualization for qualitative evaluation

**Figure 2:**
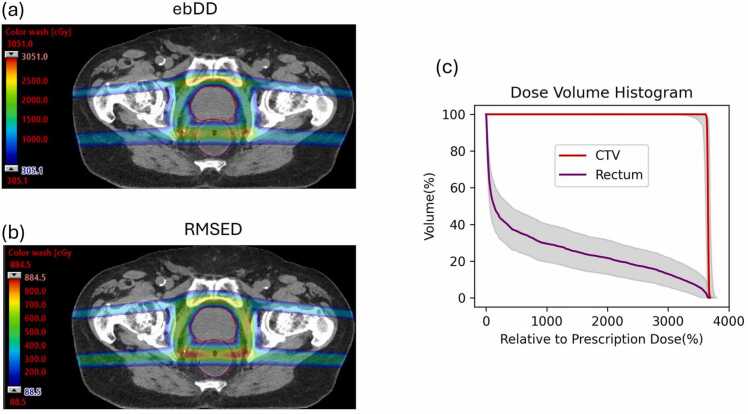
Using a prostate plan as an example, an axial slice of the 3D maps ranging from 10% to 100% of the maximum value of ebDD and RMSED are shown in (a) and (b), respectively. (c) The DVH bands in gray shade for the CTV and the rectum. Abbreviation: DVH, dose-volume histogram.

Figure 2a and 2b displays an axial slice of the 3D maps ebDD and RMSED for an example prostate plan. The qualitative visualizations of these 2 maps were very similar, with differences only in quantities due to the use of different algorithms to calculate dose variations in each voxel. Both highlight areas sensitive to setup and range uncertainties. Dose variability within the CTV is small, likely due to the robust optimization applied during treatment planning. In contrast, dose variations are more pronounced in the high-dose gradient regions just outside the CTV (eg, rectum). The DVH band for the CTV is very narrow, while the DVH band for the rectum is noticeably wider (Figure 2c). Although the DVH band does not provide spatial information, the wider band observed for the rectum aligns with the greater dose variations shown in the ebDD and RMSED maps. Conversely, the narrow DVH band for the CTV is consistent with the smaller dose variability within the target.

**Figure 3:**
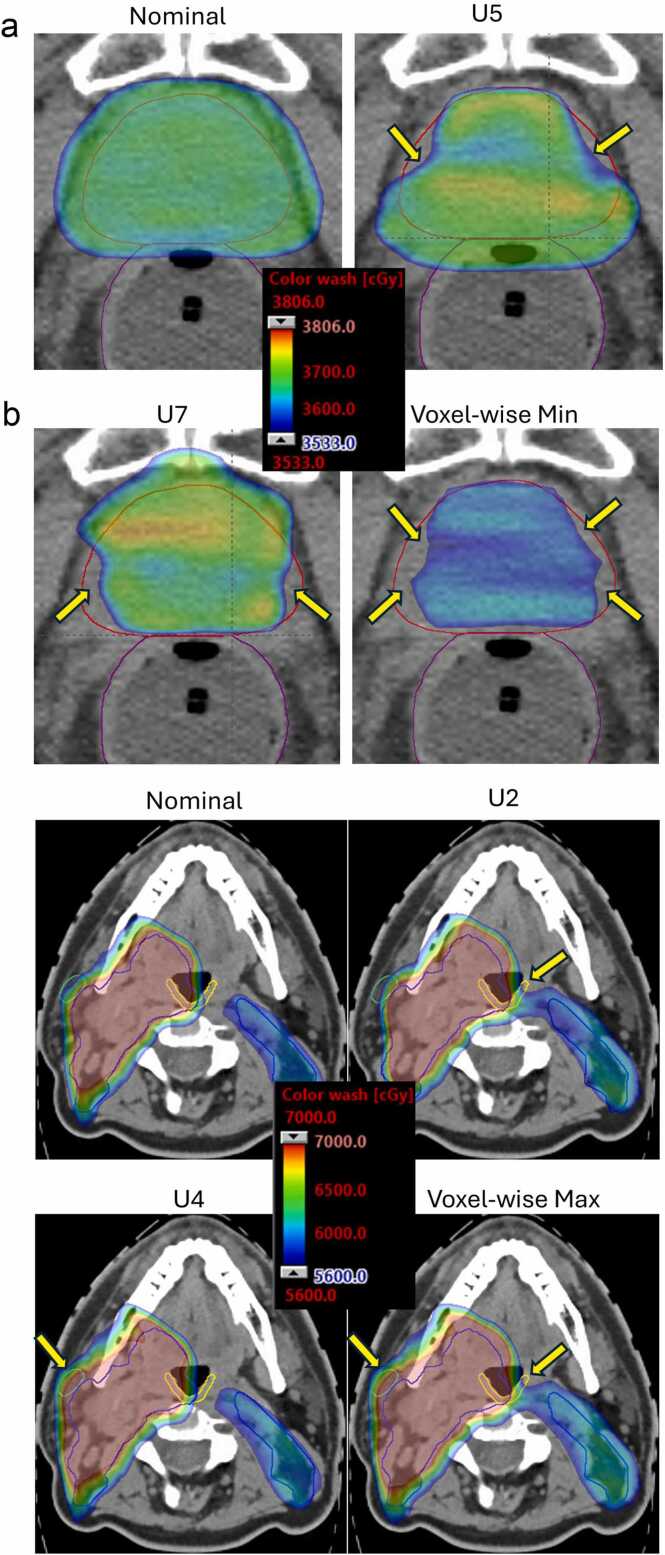
Color-washed dose distributions for (a) a representative prostate case and (b) a head and neck case, showing the nominal scenario, selected physical uncertainty scenarios, and the voxel-wise worst-case. In (a), the red contour represents the CTV (prescription dose: 3625 cGy); in (b), the blue contour represents the CTV (prescription dose: 5600 cGy), the yellow contour indicates the constrictor, and the green contour indicates the right parotid gland. Yellow arrows highlight areas where uncertainties may lead to CTV underdosing or OAR overdosing. These examples demonstrate that physical scenario worst-case evaluation requires reviewing each region of interest and metric individually, as the worst scenario may differ. In contrast, the voxel-wise worst-case identifies all potential areas of concern in a single distribution.

Figure 3a and 3b shows the color-washed dose distributions for the nominal scenario, selected physical uncertainty scenarios, and the voxel-wise worst-case, for a representative prostate case and a head and neck case, respectively. In Figure 3a (a prostate case), the selected physical uncertainty scenarios (U5 and U7) each reveal underdosed regions in different anatomical locations. This means that to identify all areas of potential concern using physical scenarios, each scenario must be reviewed individually. In contrast, the voxel-wise worst-case distribution consolidates the worst dose values from all scenarios into a single map, enabling simultaneous visualization of all potentially underdosed regions. Similarly, in Figure 3b (a head and neck case), different physical scenarios produce the worst-case dose for different OARs: the U2 scenario (setup uncertainty of (3 mm, 0, 0) in the L/R, A/P, S/I directions and a range uncertainty of −3.5%) resulted in the highest mean dose for constrictor (31.1 Gy vs 28.6 Gy nominal) but a lower mean dose to the right parotid (18.8 Gy vs 21.7 Gy), while the U4 scenario (setup uncertainty of (−3, 0, 0 mm) in the L/R, A/P, S/I directions and a range uncertainty of −3.5%) gave the highest dose to the right parotid (27.8 Gy vs 21.7 Gy) and a lower mean dose to the constrictor (28.2 Gy vs 28.6 Gy) compared to the nominal scenario. These examples illustrate that, when using the physical scenario worst-case method, each ROI and evaluation metric must be assessed individually, as the corresponding worst physical scenario may differ. In contrast, the voxel-wise worst-case method captures the worst dose distribution for all OARs simultaneously, allowing potential high-risk regions for all OAR structures to be visualized in a single distribution.

## Discussion

In this study, we evaluated the robustness of 20 clinical IMPT plans (10 prostate and 10 head and neck) using commonly adopted setup and range uncertainty settings, as well as widely used worst-case approaches. We assessed several methods within the worst-case framework, including ebDD, RMSED, voxel-wise worst-case, physical scenario worst-case, and DVH band. By directly comparing different worst-case robustness evaluation methods for each patient, we aim to provide insights into an effective and practical approach for robustness evaluation.

As expected from the inherent definitions of the methods, our correlation analysis clearly grouped the ebDD and RMSED together, while the voxel-wise worst-case and physical scenario worst-case formed another group. Robustness evaluation methods within each group showed strong correlations, but little correlation was observed between the groups. This can be explained by the fact that the 2 groups assess different aspects of the plan. Specifically, ebDD and RMSED evaluate the variation across scenarios, while voxel-wise worst-case and physical scenario worst-case focus on worst-case DVH-point data. The DVH band, a 1-dimensional summary of dose variations across physical scenarios that also captured worst-case DVH evaluation metrics, serves as a bridge between the 2 groups and demonstrated moderate correlation with both.

For quantitative DVH-point evaluation, our analysis shows that the voxel-wise worst-case yielded more conservative values. This is expected, as the voxel-wise worst-case represents the worst value for each voxel, making it inherently more conservative than the physical scenario worst-case method. This observation is consistent with the findings of Korevaar et al,^2^ who reported that corrections of approximately −0.9% for D98 and +2.3% for D2 were needed when using voxel-wise minimum and maximum values for plan evaluation relative to historical PTV-based metrics. This suggests that DVH data extracted from voxel-wise worst-case distributions may be overly conservative. In contrast, the worst DVH values derived from the DVH band of physical uncertainty scenarios may provide a more balanced and clinically appropriate approach for dosimetric quantitative evaluation. The DVH band allows for the assessment of DVH points by verifying whether the worst DVH band border meets the plan evaluation criteria. It also provides a simple way to visualize the extent of DVH variation, reflecting the plan's sensitivity to uncertainties such as setup and range errors. However, the DVH band lacks spatial information, making it impossible to identify the specific locations of potential underdosing or overdosing.

For qualitative analysis through 3D visualization, the ebDD and RMSED methods provide information into areas with significant dose variations due to uncertainties. However, large dose variations in specific ROIs do not necessarily indicate a clinically meaningful impact. This is particularly relevant for parallel structures, where high AUC values of ebDD or RMSED may not correlate with significant clinical consequences. For example, if the ebDD or RMSED analysis reveals substantial dose variation in the parotid gland, it does not automatically imply high risk, especially if the parotid receives a low overall dose. On the other hand, evaluating the 3D dose distribution using either the physical uncertainty scenarios or voxel-wise worst-case methods can help identify areas of potential underdosing in the CTV or overdosing in the OARs. However, since the physical worst-case scenario varies for different ROIs and evaluation metrics, each must be assessed individually when using the physical scenario worst-case method. In contrast, the voxel-wise worst-case method captures the worst dose distribution for all evaluation metrics, allowing the identification of potential areas of concern with a single dose distribution.

When evaluating a plan, it is typically assessed quantitatively using DVH-point evaluation metrics and qualitatively by reviewing the 3D dose distribution to identify areas of underdosing or overdosing. This same approach should apply to robustness evaluation. For example, a potential robustness evaluation strategy could combine the strengths of both the DVH band and the voxel-wise worst-case dose distribution: using the DVH band to assess whether the worst DVH band boundary meets the plan evaluation criteria, and, if concerns are identified for any specific metric, employing the voxel-wise worst-case dose distribution to pinpoint the areas of potential concern. Although this study focuses on pretreatment plan robustness evaluation, the proposed evaluation methods should also be applicable to verification plans recomputed on verification/QA CTs for on-treatment plan robustness evaluation.

It is important to acknowledge that the worst-case approach assumes systematic setup and range errors, which are commonly used in clinical practice and reflect a conservative approach. However, by not accounting for random setup errors across multiple fractions, there is a risk of an overly conservative assessment of robustness. A more conservative approach to target coverage can sometimes negatively affect normal tissue sparing. Ideally, robustness evaluations would involve identifying each source of uncertainty, assessing the extent and distribution of each uncertainty, and simulating the effects by considering both random and systematic components, using the appropriate distribution for each uncertainty. In practice, however, this process is computationally and resource intensive. Therefore, there is a need for a simple yet effective method for plan assessment in daily clinical practice. Studies^22^, ^23^ compared the worst-case approach with more comprehensive Monte Carlo-like statistical approaches and concluded that the worst-case approach is a fast and adequate method for clinical use.

While robustness evaluation is increasingly being adopted in particle therapy clinical practice, many clinical trials—especially earlier ones—still rely on the PTV concept developed for photon therapy, which does not fully account for particle-specific uncertainties. Recently, there has been a growing demand and initiative to transition particle therapy clinical trials toward robustness evaluation. This study offers timely insights to help inform the choice of a more appropriate, practical, and effective assessment strategy. However, widespread implementation of consistent robustness evaluation strategies requires improved support from commercial vendors. Currently, robustness evaluation tools, particularly robustness reporting features in TPSs, lack the flexibility and functionality needed. In this study, for example, we relied on in-house software to implement the recommended evaluation methods. For multi-institutional clinical trials, standardized and reproducible vendor-supported tools are essential. Bridging this gap will require close collaboration among researchers, clinicians, and industry partners. It is critical that the proton therapy community conducts studies like this one and clearly communicates practical needs to vendors, helping to transform research findings into tools that enable widespread adoption in clinical practice.

## Conclusion

Different robustness evaluation methods offer different types of information. Our study provides valuable insights to help identify an effective and practical approach for clinical practice. Based on our findings, we propose a potential evaluation strategy: use the DVH band derived from physical uncertainty scenarios to assess whether the worst boundary values meet plan evaluation criteria, and, when concerns arise, apply the voxel-wise worst-case dose distribution to localize areas of potential risk.

## Funding

None.

## Author Contributions

Xiaoying Liang: Conceptualization, Data analysis, Writing- Original draft. Chunbo Liu: Data collection, Software. Niles Zhang: Literature review, Data analysis. All authors contributed to clinical expertise and Writing- Review and Editing.

## Declaration of Conflicts of Interest

The authors declare no conflict of interest.

## Declaration of generative AI and AI-assisted technologies in the writing process

During the preparation of this work, the authors used ChatGPT in order to correct grammar errors. After using this tool/service, the authors reviewed and edited the content as needed and take full responsibility for the content of the publication.

## IRB

This study was granted IRB exemption by the Mayo Clinic Institutional Review Board (IRB, No.: 22-009186).

## Data Availability

The data that support the findings of this study are available from the corresponding author upon reasonable request.
